# Differential susceptibility of bovine caruncular and trophoblast cell lines to infection with high and low virulence isolates of *Neospora caninum*

**DOI:** 10.1186/s13071-017-2409-9

**Published:** 2017-10-10

**Authors:** Laura Jiménez-Pelayo, Marta García-Sánchez, Javier Regidor-Cerrillo, Pilar Horcajo, Esther Collantes-Fernández, Mercedes Gómez-Bautista, Nina Hambruch, Christiane Pfarrer, Luis Miguel Ortega-Mora

**Affiliations:** 1 SALUVET, Animal Health Department, Complutense University of Madrid, Ciudad Universitaria s/n, 28040 Madrid, Spain; 2 Department of Anatomy, University of Veterinary Medicine Hannover, Bischofsholer Damm 15, 30173 Hannover, Germany

**Keywords:** *Neospora caninum*, Cattle, Isolates, Virulence, Placenta, Trophoblast, Caruncle, Adhesion, Invasion, Proliferation

## Abstract

**Background:**

*Neospora caninum*, one of the main causes of abortion in cattle, is very effective at crossing the placental barrier and placental damage is crucial in the pathogenesis of abortion. Bovine trophoblast and caruncular cell layers are key cellular components in the maternal-foetal interface in placentomes, playing a fundamental role in placental functionality.

**Methods:**

We studied tachyzoite adhesion, invasion, proliferation and egress of high- (Nc-Spain7) and low- (Nc-Spain1H) virulence *N. caninum* isolates in established cultures of bovine caruncular epithelial (BCEC-1) and trophoblast (F3) cells. The parasite invasion rate (pInvR) and the cell infection rate (cInfR) were determined by immunostaining plaque assay at different time points and multiplicities of infection (MOIs), respectively. In addition, tachyzoite growth kinetics were investigated using real-time PCR (qPCR) analysis and immunostaining plaque assay at different times.

**Results:**

*Neospora caninum* invaded and proliferated in both cell lines. The pInvR was higher in F3 compared to BCEC-1 cells for the Nc-Spain7 isolate (*P* < 0.05), and higher for the Nc-Spain7 than the Nc-Spain1H in F3 cells (*P* < 0.01). The cInfR was also higher in F3 cells than in BCEC-1 cells for both isolates (*P* < 0.0001), and the cInfR for the Nc-Spain7 isolate was higher than for the Nc-Spain1H isolate in both cell lines (*P* < 0.05). Tachyzoite growth kinetics showed tachyzoite exponential growth until egress at 58 hpi for both isolates in F3, whereas Nc-Spain1H showed a non-exponential growth pattern in BCEC-1. Asynchronous egress of both isolates was observed from 22 h post-infection onwards in BCEC-1. In addition, the tachyzoite yield (TY_58h_) was higher in F3 than in BCEC-1 infected by both isolates (*P* < 0.0001), highlighting better replication abilities of both parasites in F3. Nc-Spain7 showed shorter doubling times and higher TY_58h_ compared to Nc-Spain1H in F3 cells; adhesion, invasion and proliferation mechanisms were very similar for both isolates in BCEC-1.

**Conclusions:**

Our results indicate a highly similar behavior of high- and low-virulence isolates in their interactions with maternal caruncular cells and suggest an important role of foetal trophoblasts in the pathogenesis of *N. caninum* infection.

## Background

*Neospora caninum* is an apicomplexan protozoan parasite, phylogenetically related to *Toxoplasma gondii*. This parasite is considered a major cause of reproductive failure in cattle worldwide [1–3], resulting in great economic losses [4]. Infection in cattle may occur through horizontal transmission, when cattle ingest sporulated oocysts shed by a canid definitive host, or by endogenous congenital transmission, from a persistently infected dam to a foetus [5]. Oral infection or recrudescence in a pregnant cow can result in abortion, birth of a weak calf or birth of a clinically healthy but persistently infected calf [1].

*Neospora caninum* is one of the most efficiently transplacentally-transmitted organisms in cattle [5]. During natural infections, invasion of the placenta, proliferation and dissemination to the foetus are crucial events in the pathogenesis of bovine neosporosis and are related to the interactions of tachyzoites with host cells and its relationship with the local immune response at the maternal-foetal interface [6]. In vivo studies demonstrated that *N. caninum* is able to infect the maternal caruncular septum before crossing to the foetal placental villus [7, 8]. Despite the fact that the placenta is directly involved in the pathogenesis of the disease [9, 10], the mechanisms by which *N. caninum* infects the placenta and reaches the fetus are poorly understood [11]. One reason could be the placental diversity [12], which makes an extrapolation of findings from one species to the other difficult. To date, only one limited in vitro study investigating the potential involvement of bovine trophoblast in *N. caninum* infection has been published [13]. In addition, no information is available regarding in vitro infection in bovine caruncular epithelial cells and the role of placental cell layers in vertical transmission.

In addition, a key question in bovine neosporosis is the influence of the parasite intra-specific variability on the outcome of infection. The lytic cycle of *N. caninum* and other apicomplexan parasites comprises the processes of invasion, adaptation to intracellular conditions, proliferation, and egress from host cells [6, 14, 15]. This sequence of events is required for parasite survival and propagation in the course of animal infection. Our previous findings demonstrated that *N. caninum* isolates of bovine or canine origin show a large biological diversity, despite being genetically similar [16]. Moreover, differences found in the events of the lytic cycle among several *N. caninum* isolates in vitro are correlated with differences observed in virulence and vertical transmission in animal models [16, 17]. Specifically, pregnant heifers inoculated at day 70 of gestation with the low-virulence isolate Nc-Spain1H spared the foetus [18], whereas foetal death occurred in all inoculated cattle with the highly virulent isolate Nc-Spain7 [19, 20].

There is no information concerning the kinetics of events in the placenta that lead to an understanding of how the parasite actually reaches the foetal tissues. The influence of biological variability of the isolate on placental damage is also poorly understood. The cow possesses a cotyledonary [21] and synepitheliochorial placenta [22], where foetal cotyledons interdigitate with maternal caruncles to form placentomes [23–25]. The trophoblast (epithelial surface of the foetal cotyledons) consists of uninucleated and binucleated cells. Binucleated cells are responsible for a “restricted” trophoblast invasion [26], playing an important role in embryo implantation and successful pregnancy outcomes. Caruncular epithelial cells form a polarized barrier that the parasite encounters before reaching and multiplying in foetal tissues. Hence, the aim of this study was to investigate the interaction of two isolates of *N. caninum* with maternal and foetal bovine target cells. Here, we studied tachyzoite adhesion, invasion, proliferation and egress of high- (Nc-Spain7) and low- (Nc-Spain1H) virulence isolates in established cultures of bovine caruncular epithelial (BCEC-1) and trophoblast (F3) cells. Since BCEC-1 and F3 cells conserve some of the properties from their tissues of origin [24, 27, 28], they are a useful tool to evaluate critical factors involved in placental pathogenesis, such as the mechanisms used by *N. caninum* to cross the placental barriers.

## Methods

### Parasites and cell cultures

Nc-Spain7 and Nc-Spain1H isolates were obtained from healthy, congenitally infected calves [29, 30] and extensively characterized using in vitro, murine and bovine models [16, 18, 20, 29, 31, 32]. Tachyzoites were routinely maintained in a monolayer culture of the MARC-145 monkey kidney cell line as described previously [16]. The *N. caninum* isolates used in this study were subjected to a limited number of culture passages (from 8 to 15) to ensure the maintenance of their in vivo biological behaviour and avoid their adaptation to the host cells [33].

A bovine trophoblast cell line F3 [28] and a bovine caruncular cell line BCEC-1 [23] were isolated from two BVD-free, pregnant cattle (*Bos taurus*) with an estimated gestational age of 5 and 4 months, respectively. Cells were grown as indicated by Hambruch et al. [28]. Briefly, cells were maintained in Dulbecco’s Modified Eagle Medium (DMEM)/Ham’s F12 containing 10% foetal calf serum (FCS) checked for the absence of specific IgG against *N. caninum* by IFAT, 100 IU/ml Penicillin, 100 mg/ml Streptomycin and 2 mM Glutamine. All experiments were carried out with cells below passage 27, when both cell lines maintained their morphological and functional features [24, 27, 28].

Tachyzoites used for in vitro assays were recovered from 2.5–3 day growth cultures of MARC-145, when the majority of the parasites were still intracellular, and purified using Disposable PD-10 Desalting Columns (G.E. Healthcare, Buckinghamshire, UK) as previously described [16]. Tachyzoite viability was checked by trypan blue exclusion. F3 and BCEC-1 cell monolayers were inoculated within 1 hour of tachyzoite collection from flasks. All in vitro experiments in F3 and BCEC-1 cell lines were assayed in quadruplicate, and two independent experiments were carried out.

### Parasite invasion rate

Parasite invasion rate (pInvR) was defined as the number of tachyzoites invading the host cell at different time-points (hours) post-infection (hpi) and were determined following the methodology described in Dellarupe et al. [17] with minimal modifications. In order to obtain a confluent monolayer of F3 and BCEC-1, cells were seeded with 2 × 10^5^ and 3 × 10^5^ cells per well, respectively. Different density of both cell types were used because F3 cells are bigger than BCEC-1 cells and formed a monolayer composed of polygonal cells while as BCEC-1 are smaller and they tended to form colonies and did not spread out the entire surface of the well. A total of 100 purified tachyzoites of each isolate were added to 24-well culture plates. Cultures were washed three times with phosphate buffered saline (PBS) at different time points (1, 2, 4, 6 and 8 hpi) for removing non-adhered/non-invading tachyzoites. Unwashed cultures were also included in the study. All plates were fixed at 48 hpi, and the pInvR was determined using single immunofluorescence staining as described below. To determine the pInvR, events (medium and large parasitophorous vacuoles) present in each well were counted using an inverted fluorescence microscope (Nikon Eclipse TE 200, Chiyoda, TYO, Japan) at a magnification of 200×. The pInvR at 1, 2, 4, 6 and 8 hpi (pInvR_1h_, pInvR_2h,_ pInvR_4h,_ pInvR_6h,_ pInvR_8h_, respectively) was determined as the number of events per well in cell monolayers washed at different time points, and the total parasite invasion rate (pInvR_T_) was determined as the number of events per well in unwashed cultures.

### Cell infection rate

Multiplicity of infection (MOI) was defined as the ratio of the number of tachyzoites added to a known number of cells in a culture. Cell infection rate (cInfR) was defined as the percentage of cells infected using different MOIs (1, 2, 4, 6, 8 and 10). Cells were cultured in 24-well plates at concentration of 2 × 10^5^ and 3 × 10^5^ cells per well for F3 and BCEC-1 cells, respectively. Infected cells were washed 3 times with PBS after 4 hpi to facilitate the synchronization of the cultures. Finally, cells were fixed at 48 hpi and stained using single immunofluorescence staining as described below.

The overall number of cells, the number of infected cells and the number of cells containing more than one vacuole (multi-infected cells) were counted in 10 arbitrarily selected fields using an inverted fluorescence microscope (Nikon Eclipse TE 200, Chiyoda, TYO, Japan) at a magnification of 200×. Counting of events was carried out on images taken with three different filters (white light for discrimination of cell limits, blue-DAPI for visualization of the nuclei and red-Alexa 594 for examination of the tachyzoites) using a Nikon DSL1 camera (Chiyoda, TYO, Japan) and overlaid using Photoshop® software (Adobe Systems Incorporated, Mountain View, CA, USA). A mean value of 50 cells was counted in each field.

### Adhesion-invasion assay

An adhesion-invasion assay was performed in F3 and BCEC-1 cultures seeded at concentration of 2 × 10^5^ and 3 × 10^5^ cells per well, respectively. Cells were infected at a MOI of 2, and cultures were washed with PBS at 4 hpi to remove non-adherent extracellular tachyzoites. Cultures were immediately fixed and double immunofluorescence staining was carried out following the protocol described below. Adhered extracellular tachyzoites (green- and red-stained) and intracellular tachyzoites (red-stained only) were counted using a fluorescence microscope (Nikon Eclipse TE 200, Chiyoda, TYO, Japan) at a magnification of 400×. A total of 1000 tachyzoites was counted in each coverslip. The percentage of intracellular tachyzoites (red-stained) respect to the total number of tachyzoites (intracellular and extracellular adhered tachyzoites) (green-stained) at 4 hpi was calculated.

### Intracellular proliferation assays: Proliferation kinetics, doubling time and tachyzoite yield determinations

Proliferation kinetics of Nc-Spain7 and Nc-Spain1H isolates in F3 and BCEC-1 cells were determined by quantifying the number of tachyzoites at specific times (4, 10, 22, 34, 46, 58, 70 and 82 hpi) by real-time PCR (qPCR). Cells were cultured and infected as indicated above using a MOI of 2. Cultures were washed at 4 hpi and subsequently maintained at 37 °C in 5% CO_2_. The samples were collected adding 200 μl of PBS, 180 μl of lysis buffer and 20 μl of proteinase K (Qiagen, Hilden, Germany) to each well at 4, 10, 22, 34, 46, 58, 70 and 82 hpi, transferred to a microcentrifuge tube and frozen at -80 °C prior to DNA extraction.

In parallel, replicates of cell cultures in coverslips were infected as described above and were labelled using double-inmunostaining to study microscopically the proliferation kinetics of both isolates in F3 and BCEC-1 cells. Three coverslips were photographed for each condition using an inverted fluorescence microscope (Nikon Eclipse TE 200, Chiyoda, TYO, Japan).

The doubling time (T_d_) was defined as the period of time required for a tachyzoite to duplicate during the exponential multiplication period, excluding the lag phase (period without parasite multiplication) and the egress phase [16]. The T_d_ was determined by applying non-linear regression analysis and an exponential growth equation using GraphPad (San Diego, CA, USA). We represented T_d_ for each isolate and each cell line as the average value obtained from all the determinations that revealed a linear regression, R^2^ ≥ 0.95.

The tachyzoite yield (TY_58h_) was defined as the average value of the number of tachyzoites quantified by qPCR at 58 hpi for each isolate and cell line.

### Immunofluorescence staining

Single immunofluorescence staining was carried out as specified previously [17] with minimal variations. Parasites in fixed cultures were stained using hyperimmune rabbit antiserum directed against *N. caninum* tachyzoites (1:1000) as a primary antibody and a 1:1000 dilution of goat anti-rabbit IgG conjugated to Alexa Fluor® 594 (red, Thermo Fisher Scientific, Waltham, MA, USA) as a secondary antibody. The nuclei were stained by washing the cells with a solution of 1:5000 DAPI in PBS.

Double immunofluorescence staining was carried out following the protocol described by Regidor-Cerrillo et al. [16] with minimal modifications. Fixed plates were treated with 3% BSA in PBS for 30 min at 25 °C to block unspecific antibody binding and autofluorescence. Then, cultures were treated with a 1:1000 dilution of anti-tachyzoite hyperimmune rabbit antiserum and a 1:1000 dilution of goat anti-rabbit IgG conjugated to Alexa Fluor® 488 (green, Thermo Fisher Scientific, Waltham, MA, USA). After this step, only extracellular tachyzoites were labelled in green. After the first staining, cells were permeabilized using a solution of 0.25% Triton 100X in PBS 0.3% BSA (30 min, 37 °C). Later, cultures were treated again with a dilution of anti-tachyzoite hyperimmune rabbit antiserum as primary antibody (1:1000) and a 1:1000 dilution of goat anti-rabbit IgG conjugated to Alexa Fluor® 594 as secondary antibody (red, Thermo Fisher Scientific, Waltham, MA, USA). Therefore, intracellular tachyzoites were labelled only in red, while extracellular tachyzoytes resulted labelled in green and in red. The nuclei were stained by washing the cells with a solution of 1:5000 DAPI in PBS and the coverslips were embedded in Fluoroprep (BioMerieux, Marcy-l’Étoile, France).

### DNA extraction and real-time PCR

Genomic DNA was extracted from cellular samples using the DNeasy® Blood & Tissue Kit (Qiagen, Hilden, Germany) according to the manufacturer’s instructions. Genomic DNA was eluted in a volume of 60 μl of molecular-grade water. Concentrations of DNA were determined for each sample using a nanophotometer (Nanophotomer®, Implen GmbH, Munich, Germany) and samples were diluted 1:4 in molecular-grade water. Quantification of *N. caninum* DNA was performed by real-time PCR using an Applied Biosystems 7300 Real-Time PCR System (Applied Biosystems, Foster City, CA, USA). The Nc-5 region was targeted as described elsewhere [34]. Five μl of diluted DNA from each sample were used for the qPCR assays. The number of *N. caninum* tachyzoites was determined by interpolating the C_t_ values (cycle threshold value, which represents the fractional cycle number reflecting a positive PCR result) on a standard curve. The standard curve was designed for the quantification of 10^-1^–10^4^ tachyzoites according to Regidor-Cerrillo et al. [16]. To normalize the quantification of the parasites in each sample, a bovine β-actin standard curve was designed (from 64 ng of DNA per μl to 0.2 ng per μl). The results were expressed as the relation between amounts of parasite DNA and cell DNA (R^2^ ≥ 0.99; slope values varied from -3.67 to -3.13).

### Statistical analysis

The parametric one-way ANOVA test, followed by a Tukey’s multiple comparisons test, was performed to investigate the influence of time on the pInvR and MOI in the cInfR, and the two-way ANOVA test, followed by a Tukey’s multiple comparisons test, was performed to study the influence of the parasite isolate and the cell type on the pInvR and cInfR. A Chi-square test was carried out to investigate the differences in the percentages of intracellular tachyzoites at 4 hpi in both target cell types. Bonferroni correction was used to eliminate error associated with making multiple comparisons. Statistical significance was established as *P* < 0.05. Differences that showed *P*-values ≥ 0.05 and < 0.1 were considered to be trending towards statistical significance. GraphPad Prism 5 v.5.01 (San Diego, CA, USA) software was used to perform all statistical analyses and graphical illustrations.

## Results

### Parasite invasion rate (pInvR)

**Fig. 1:**
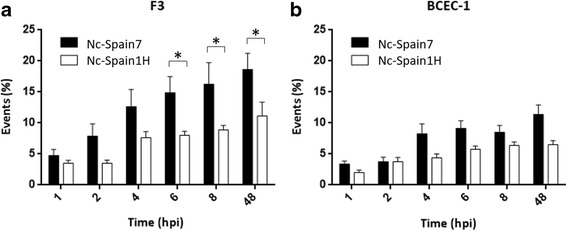
Parasite invasion rates in F3 and BCEC-1 cells infected by Nc-Spain7 and Nc-Spain1H isolates. Graphs represent parasite infection rates in F3 (**a**) and BCEC-1 cells (**b**) defined as the percentage of invaded tachyzoites (number of events per well) studied at different time points for Nc-Spain7 and Nc-Spain1H. Each column and error bar represents the mean and the SD of 4 replicates from 2 independent assays at the indicated sampling times. The total number of invaded tachyzoites was determined by single immunofluorescence staining of events (parasitophorous vacuoles and lysis plaques) followed by counting using an inverted fluorescence microscope. Significantly higher pInvRs were found in F3 cells compared to BCEC-1 cells infected with Nc-Spain7 (*P* < 0.01), whereas no differences were found in the pInvRs of F3 and BCEC-1 cells infected by Nc-Spain1H (*P* > 0.05). * represents significant differences between isolates

To investigate the impact of the placental cell type in parasite invasion, the pInvR was evaluated in trophoblasts and caruncular cells at different time points post-infection (1, 2, 4, 6 and 8 hpi). The pInvRs of the Nc-Spain7 and Nc-Spain1H isolates in F3 and BCEC-1 cells are shown in Fig. 1. The number of invaded tachyzoites for both isolates significantly increased until 4 hpi in both F3 (Nc-Spain7, ANOVA: *F*_(5,35)_ = 11.87, *P* < 0.0001; Nc-Spain1H, ANOVA: *F*_(5,35)_ = 7.211, *P* < 0.0001, followed by a Tukey’s multiple comparisons test) and BCEC-1 cells (Nc-Spain7, ANOVA: *F*_(5,35)_ = 9.825, *P* < 0.0001; Nc-Spain1H, ANOVA: *F*_(5,35)_ = 9.156, *P* < 0.0001, followed by a Tukey’s multiple comparisons test). From 4 hpi onwards, significant differences were not observed.

Regarding the influence of the target cell type, a higher pInvR was observed in F3 cells compared to BCEC-1 cells from 6 hpi onwards for the Nc-Spain7 isolate (two-way ANOVA test: *F*_(3,168)_ = 27.25, *P* < 0.0001, followed by a Tukey’s multiple comparisons test). No statistically significant differences were found when the pInvRs of Nc-Spain1H in both cell lines were investigated.

The influence of the parasite isolate on the invasion of bovine trophoblasts and caruncular cells was also investigated by comparison of the pInvRs between the Nc-Spain1H and Nc-Spain7 isolates, assayed at different times of infection. Nc-Spain7 showed a pInvR significantly higher than Nc-Spain1H from 6 hpi onwards in F3 cells (two-way ANOVA test: *F*_(3,168)_ = 27.25, *P* < 0.0001 followed by a Tukey’s multiple comparisons test) (Fig. 1a). However, no statistically significant differences in pInvR were found between isolates in BCEC-1 cells (Fig. 1b).

### Cell infection rate (cInfR)

The percentage of infected cells (cInfR) and the percentage of multi-infected cells were evaluated at different MOIs.

**Fig. 2:**
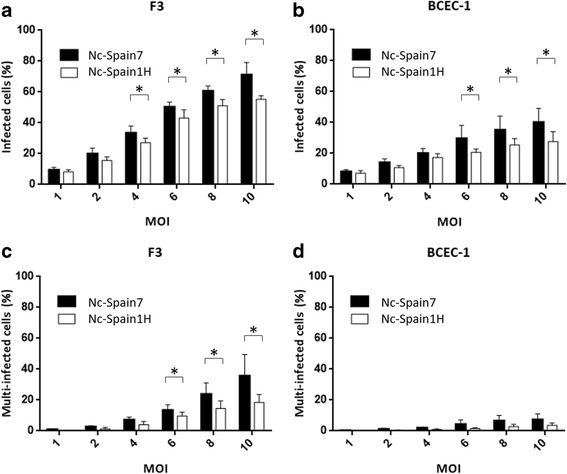
Infection and multi-infection rates in F3 and BCEC-1 cells infected by Nc-Spain7 and Nc-Spain1H isolates. Graphs represent the cell infection rates as the percentage of infected cells in F3 (**a**) and BCEC-1 cells (**b**) for both isolates and the percentage of cells with multi-infection (more than one parasitophorous vacuole) in F3 (**c**) and BCEC-1 cells (**d**). Each column and error bar represents the mean and the SD of 4 replicates from 2 independent assays using different MOIs. The total number of cells, the number of infected cells and the number of cells with multi-infection were determined by double immunofluorescence staining followed by counting using an inverted fluorescence microscope. The cInfRs were higher in F3 than in BCEC-1 cells infected by both isolates (*P* < 0.0001). The percentage of cells containing more than a single vacuole was also higher in F3 than in BCEC-1 cells infected by both isolates (*P* < 0.05). * represents significant differences between isolates

The number of infected cells significantly increased with increasing MOIs in both cell lines F3 (Nc-Spain7, ANOVA: *F*_(5,42)_ = 228.5, *P* < 0.0001; Nc-Spain1H, ANOVA: *F*_(5,42)_ = 273.4, *P* < 0.0001, followed by a Tukey’s multiple comparisons test) and BCEC-1 (Nc-Spain7, ANOVA: *F*_(5,42)_ = 30.04, *P* < 0.0001; Nc-Spain1H, ANOVA: *F*_(5,42)_ = 42.60, *P* < 0.0001, followed by a Tukey’s multiple comparisons test). The cInfRs were higher in infected F3 than in BCEC-1 cells for both Nc-Spain7 and Nc-Spain1H isolates at the same MOI (Fig. 2a, b) (two-way ANOVA test: *F*_(3,168)_ = 222.4, *P* < 0.0001, followed by a Tukey’s multiple comparisons test). The percentage of cells containing more than a single vacuole (Fig. 2c, d) was also higher in F3 than in BCEC-1 infected by both isolates (two-way ANOVA test: *F*_(3,168)_ = 93.64, *P* < 0.0001, followed by a Tukey’s multiple comparisons test).

In addition, Nc-Spain7 showed a higher cInfR than Nc-Spain1H in both cell lines at different MOIs (two-way ANOVA test: *F*_(3,168)_ = 222.4, *P* < 0.0001, followed by a Tukey’s multiple comparisons test) (Fig. 2a, b). We also observed that Nc-Spain7 showed a higher percentage of multi-infected cells than Nc-Spain1H in F3 cells at 6, 8 and 10 MOIs (two-way ANOVA test: *F*_(3,168)_ = 93.64, *P* < 0.0001, followed by a Tukey’s multiple comparisons test) (Fig. 2c). However, no significant differences in the number of multi-infected cells were found between isolates in BCEC-1 cells (Fig. 2d).

### Adhesion-invasion assay

**Fig. 3:**
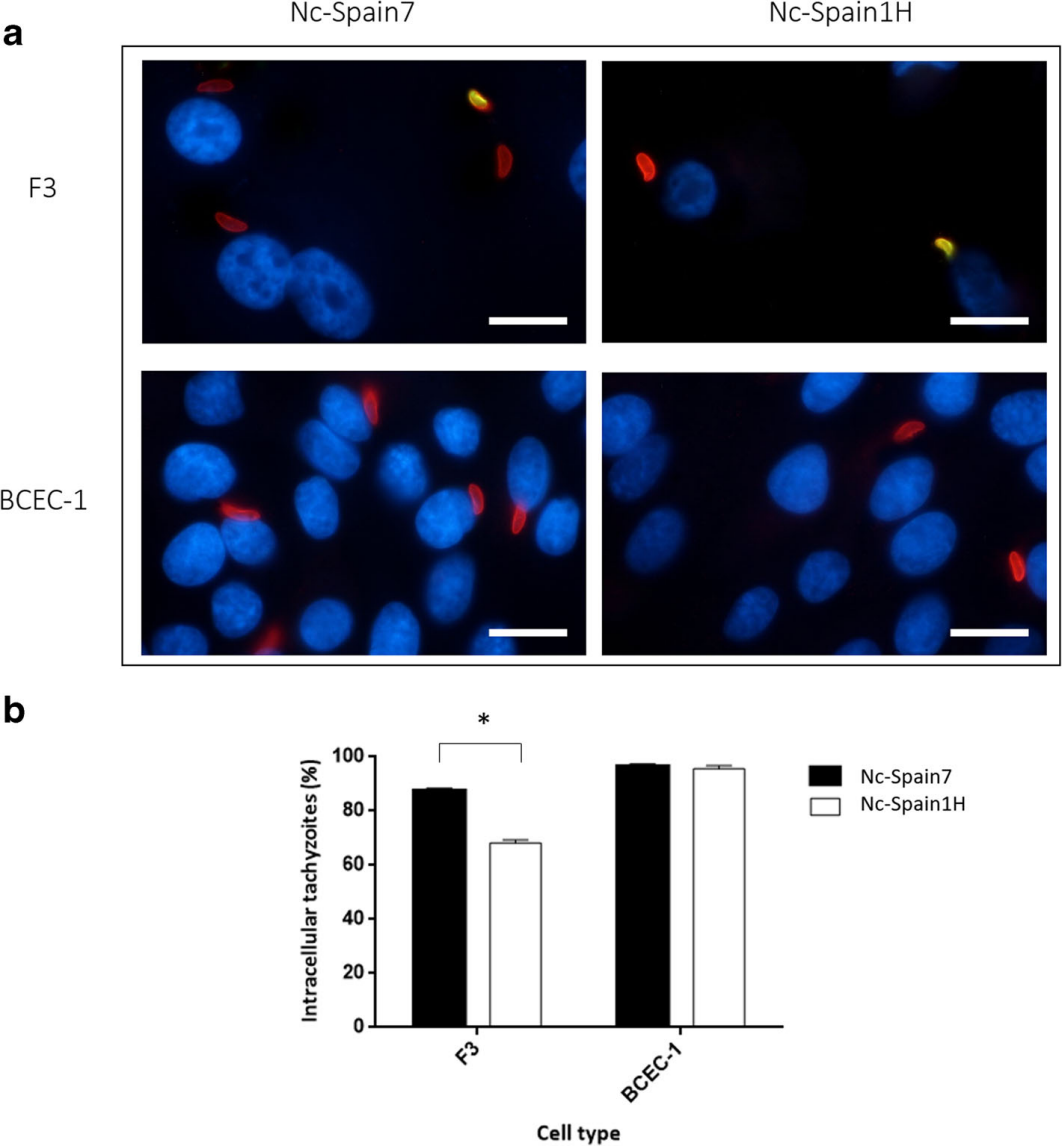
Adhesion assay in F3 and BCEC-1 cells infected by Nc-Spain7 and Nc-Spain1H at 4 hpi. Double immunofluorescence staining was performed, and adhered extracellular tachyzoites were stained with Alexa Fluor® 488 (*green*) and Alexa Fluor® 594 (*red*), whereas intracellular tachyzoites were stained with Alexa Fluor® 594 (*red)*. Nuclei were stained with DAPI (*blue*). Tachyzoites were counted in 10 arbitrarily selected fields, and the percentage of intracellular tachyzoites relative to the number of total adhered tachyzoites at 4 hpi was calculated. Representative images at a magnification of 1000× (**a**) show the adhesion assay performed in F3 and BCEC-1 cells infected with both isolates. The graph (**b**) represents the percentage of intracellular tachyzoites of Nc-Spain7 and Nc-Spain1H relative to the total number of intra- and extracellular tachyzoites adhered to F3 and BCEC-1 cells. Each column and error bar represents the mean and the SD of 4 replicates from 2 independent assays. BCEC-1 cells showed a significantly higher percentage of intracellular tachyzoites than F3 cells (*P* < 0.0001, Chi-square test). The percentage of intracellular tachyzoites for Nc-Spain7 (88%) was significantly higher than for Nc-Spain1H (69%) in F3 (*P* < 0.0001), whereas the percentage of intracellular tachyzoites of both isolates in BCEC-1 was the same (96%). * represents significant differences between isolates. *Scale-bars*: **a**, 40 μm

In the light of the differences in pInvR and cInfR between both cells lines, an adhesion-invasion assay was performed to investigate whether these differences could be attributed to a different adhesion ability of the tachyzoites in these two cell lines, a different ability to penetrate in the cells or both (Fig. 3a). In this assay, non-adhered tachyzoites were eliminated in the washing step at 4 hpi before the fixation and extra- and intracellular adhered tachyzoites were counted. The percentage of intracellular tachyzoites respect to the total adhered intra- and extracellular tachyzoites was calculated.

Surprisingly, both isolates showed that almost 100% of adhered tachyzoites were intracellular at 4 hpi in BCEC-1 cells, whereas a minor percentage of intracellular tachyzoites was observed in F3 cells, 88 and 69% for Nc-Spain7 and Nc-Spain1H, respectively. A significantly higher number of adhered tachyzoites from both isolates were internalized in BCEC-1 cells than in F3 cells at 4 hpi (Chi-square test: *χ* ^2^ = 287.6, *df* = 3, *P* < 0.0001) (Fig. 3b).

Differences between isolates were not observed in BCEC-1, although the high-virulence isolate Nc-Spain7 showed a better ability to penetrate than the low-virulence isolate Nc-Spain1H in F3 cells (Fisher’s exact test: *P* < 0.0001) (Fig. 3b).

### Proliferation kinetics, doubling time comparisons and tachyzoite yield determination

**Fig. 4:**
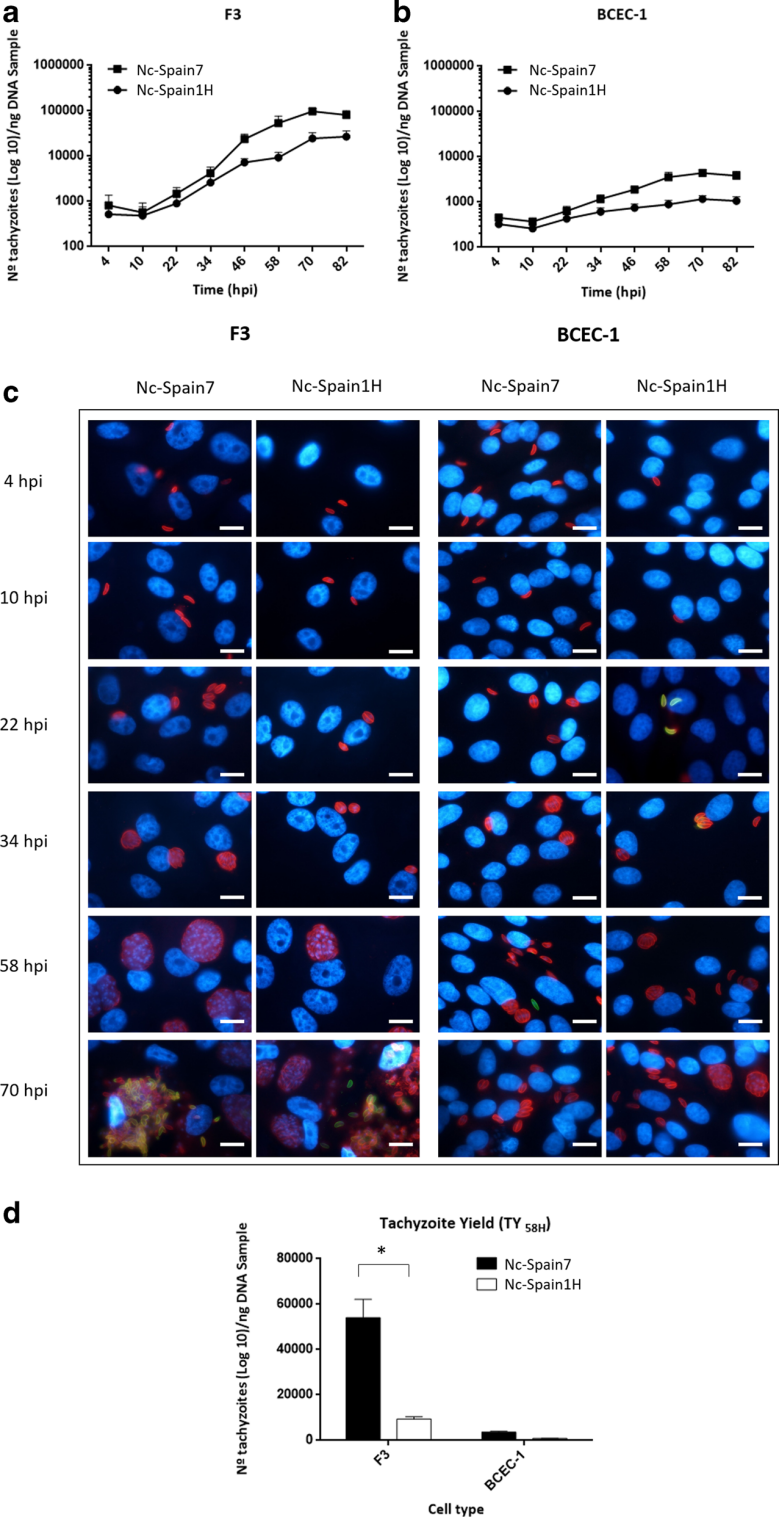
Proliferation kinetics over time and tachyzoite yield at 58 hpi. Graphs (**a** and **b**) represent the average number of tachyzoites for each time-point for all individual experiments with an R^2^ > 0.95, except for BCEC-1 cells infected by Nc-Spain1H, which showed a non-exponential growth pattern. Error bars indicate the SD. Representative images (**c**) show the proliferation kinetics over time of Nc-Spain7 and Nc-Spain1H isolates in F3 and BCEC-1 cultures. The bar graph (**d**) represents the tachyzoite yield at 58 hpi for Nc-Spain7 and Nc-Spain1H in F3 and BCEC-1 cells. Each column and error bar represents the mean and the SD of 4 replicates from 2 independent assays. The TY_58h_ was fifteen times higher in F3 cells than in BCEC-1 cells infected with Nc-Spain7, and ten times higher in F3 cells infected with Nc-Spain1H. Statistical differences were found in the TY_58h_ between isolates in F3 cells, with the TY_58h_ of Nc-Spain7 significantly higher than for Nc-Spain1H (*P* < 0.0001). * represents significant differences between isolates. *Scale-bars*: **c**, 10 μm

An in vitro intracellular proliferation assay was carried out to study proliferation and egress events of the lytic cycle of *N. caninum* in trophoblast and caruncular cell cultures. Proliferation kinetics over time assessed by qPCR are represented in Fig. 4a, b. The growth curves of both isolates in F3 and the growth curve of Nc-Spain7 in BCEC-1 adjusted to exponential growth from 10 hpi to 70 hpi, whereas the growth curve of Nc-Spain1H in BCEC-1 did not adjust either to the exponential or linear growth mathematical model. Analysing the T_d_, we observed a delay in the multiplication of *N. caninum* in BCEC-1 cells, with the average T_d_ value of Nc-Spain7 1.5-times higher in BCEC-1 cells (14.603 ± 1.428) than in F3 cells (9.425 ± 0.239) (one-way ANOVA: *F*_(2,21)_ = 6.966, *P* = 0.0048 followed by a Tukey’s multiple comparisons test). The Nc-Spain1H isolate showed an average T_d_ value of 12.246 ± 0.893 in F3 cells. The T_d_ value for Nc-Spain1H in BCEC-1 could not be calculated due to the lack of exponential growth of Nc-Spain1H in BCEC-1. Nevertheless, no significant differences were found in the average T_d_ values for Nc-Spain7 and Nc-Spain1H isolates in F3 cells.

A microscopic examination of cultures fixed at different time points showed that the multiplication of Nc-Spain7 and Nc-Spain1H isolates began between 10 and 22 hpi. Differences in the parasitophorous vacuole size between both isolates were observed in F3 cells from 34 hpi onwards, with bigger vacuoles in Nc-Spain7 infected cells. However, no differences between the isolates were demonstrated by immunofluorescence in BCEC-1 cells. Between 58 and 82 hpi, asynchronous rupture of host cells and egress of the tachyzoites were observed in F3 cells. However, interestingly, an early egression of tachyzoites from 22 hpi onwards was observed in BCEC-1 (Fig. 4c).

The TY_58h_ was assessed to determine the number of tachyzoites produced during the same intracellular period after invasion, prior to complete tachyzoite egress from cell cultures (Fig. 4d). The TY_58h_ was 15-times higher in F3 cells than in BCEC-1 cells infected with Nc-Spain7, and 10-times higher in F3 cells infected with Nc-Spain1H. Comparing the isolates, Nc-Spain7 showed a higher TY_58h_ than Nc-Spain1H in F3 (one-way ANOVA: *F*_(3,28)_ = 37.35, *P* < 0.0001 followed by a Tukey’s multiple comparisons test), whereas no differences in the TY_58h_ were found in BCEC-1.

The results obtained in the present work are summarized in Table 1.

**Table 1:** Summary of virulence traits as a function of cell type and *N. caninum* isolate

		pInvR^a^	cInfR^b^	% Multi-infection^c^	Invasion efficiency^d^	TY_58H_^e^	T_d_^f^
Cell type comparisons (F3 *vs* BCEC-1)	Nc-Spain7	+	++++	+	- - - -	+	- -
	Nc-Spain1H	NS^h^	++++	+	- - - -	+	NC^**g**^
Isolate comparisons (Nc-Spain7 *vs* Nc-Spain1H)	F3	++	+	++++	++++	++	NS
	BCEC-1	NS	+	NS	NS	NS	NC^**g**^

^a^pInvR (parasite invasion rate): number of tachyzoites invading the host cell at different time points post-infection

^b^cInfR (cell infection rate): percentage of cells infected using different parasite doses

^c^% Multi-infection: percentage of cells containing more than one vacuole

^d^Invasion efficiency: results from adhesion-invasion assay, percentage of intracellular tachyzoites relative to the total number of tachyzoites at 4 hpi

^e^TY_58H_ (tachyzoite yield at 58 hpi): average number of tachyzoites quantified by qPCR at 58 hpi

^f^T_d_ (Doubling time): period of time required for a tachyzoite to duplicate during the exponential multiplication period, excluding lag and egress phases

^g^ *NC* data not comparable. The T_d_ value for Nc-Spain1H isolate in BCEC-1 cells could not be calculated due to the lack of exponential growth of Nc-Spain1H in BCEC-1

^h^ *NS* no significant differences

+/++/++++ indicate higher rates of each parameter assayed with a significance of *P* < 0.05, *P* < 0.01, P < 0.0001, respectively

-/- -/- - - - indicate lower rates of each parameter assayed with a significance of P < 0.05, P < 0.01, P < 0.0001, respectively

## Discussion

In the present study, we established for the first time a species- and organ-specific in vitro model for each of the host cell layers in the maternal-foetal interface of the bovine placenta to study *N. caninum* infection. To date, several in vitro studies have been carried out using established cell lines, such as Marc-145, HeLa, BeWo or ovine trophoblast cells, to investigate the invasion and proliferation of different *N. caninum* isolates [16, 35, 36]. There is only one (limited) descriptive study concerning the interaction of the parasite with bovine trophoblast cells [13], and no data are available about the parasite’s interaction with bovine caruncular cells. The interaction between *N. caninum* and these target cells has been studied in the present work, using two isolates of different virulence and the two cell lines that represent the maternal-foetal interface. The cell lines used in this work (bovine trophoblast cells, F3, and bovine caruncular epithelial cells, BCEC-1) were isolated from fifth- and fourth-month pregnant heifers, respectively, and they have maintained at least part of their morphological and functional characteristics [24, 27, 28]. Thus, they may be a useful tool to investigate the pathways of *N. caninum* infection during transplacental transmission during the second trimester of pregnancy when the majority of abortions caused by *N. caninum* occur [1]. Investigations were focused on the lytic cycle of the tachyzoites (host-cell invasion, proliferation and egress). The processes implicated in the lytic cycle of the parasite are essential for the invasion of host tissues, the distribution of the parasite through the organism and its distribution to the placenta. As a consequence, abortion or transplacental transmission may occur [1, 6]. Here, two *N. caninum* isolates with marked differences in virulence were able to establish themselves and multiply both in the maternal ephitelium of the caruncle and in the foetal trophoblast, although differences in the infection of both cell types were found.

Our results showed a lower infection rate, as well as a lower percentage of cells with multi-infection, for both parasite isolates in BCEC-1 cells relative to F3 cells. Therefore, *N. caninum* tachyzoites more efficiently infect trophoblast cells compared to bovine caruncular epithelial cells, meaning bovine trophoblast cells are more susceptible to *N. caninum* infection. In experimental infections, higher parasite burdens and more severe lesions have been found in the foetal part of the placenta [19, 20]. As previously demonstrated, BCEC-1 is an established cell line showing in vitro characteristics of a morphologically and functionally intact epithelial barrier with apical microvilli and junctional complexes (zonula occludens, zonula adherens and desmosomes) [23, 24] as described in vivo [37, 38]. This polarized barrier, with apical junctional complexes obliterating the paracellular space, establishes an effective paracellular barrier to diffusion of fluid and solutes, limiting the passage of foetal and maternal metabolites [39]. These characteristics may be hindering the paracellular passage of *N. caninum* across the epithelium. Foetal cells (F3) share many properties with maternal cells (BCEC-1), including apical microvilli and expression of the tight junctional zonula occludens protein both in vivo [37, 38] and in vitro [28]; however, in contrast to the maternal BCEC-1 cells, mononuclear trophoblast cells have phagocytic phenotypes in vivo [40]. Also bovine F3 cells may form binucleated cells [28], which have phagocytic activity as has been previously described for trophoblast giant cells of various species [40–42]. The phagocytic activity of mono- and binucleate trophoblast cells may be mediating parasite passage to the foetus [13]. In fact, in a BALB/c mouse model infected with *T. gondii*, a higher frequency of infected placentas was observed at later stages of pregnancy, which has been correlated with a higher phagocytic efficiency of the placental tissues in this period [43]. Therefore, although differences in junctional complexes between both cell types should be investigated to evaluate their influence in parasite invasion, it seems probable that the phagocytic ability of trophoblast cells may partially explain the higher susceptibility to parasite invasion observed in these cells.

Noting the differences in the invasion and infection rates between both cell lines, an adhesion-invasion assay was performed in order to elucidate whether these differences could be associated with lower adhesion, penetration or both. Our results revealed that, contrary to expectations, both isolates, which had presented lower cell infection rate in BCEC-1 cells compared to F3 cells, showed a highly efficient invasion with almost 100% penetration of the adhered tachyzoites in BCEC-1 cells at 4 hpi. Thus, the lower invasion of *N. caninum* observed in BCEC-1 cells may be due to a lower ability to adhere or to a fragile adhesion to host-cell receptors. Tachyzoite adhesion occurs in two phases, as previously described [6]. The first step is the establishment of low-affinity contact between tachyzoites and the host-cell surface membrane, where surface antigens of *N. caninum* tachyzoites such as NcSAG1 and NcSRS2 are involved. Later, the actual adhesion process occurs via to microneme proteins (especially NcMIC3), which bind to host-cell surface chondroitin sulfates. Studying the differences in the type and abundance of superficial receptors responsible for the high-affinity interaction with tachyzoites between both cell lines could aid the understanding of the diminished adhesion of *N. caninum* in bovine caruncular cells.

Concerning the growth kinetics of *N. caninum* in foetal and maternal cells, our results showed a dramatically lower proliferation of both isolates in caruncular cells. Moreover, Nc-Spain1H did not adjust to an exponential growth in maternal cells. Differences observed between both cell types may be partially attributed to a different degree of maturation. While the foetus is not completely immunocompetent in the second trimester of gestation, maternal cells have immunocompetent abilities that may restrict the infection and proliferation of the parasite. In vivo studies have demonstrated the influence of the gestational stage on the outcome of *N. caninum* infection [44–47]. It is known that the survival of the foetus depends on the state of development of its immune system, as higher abortion rates, higher parasite burdens and more severe lesions were observed in foetal tissues when infection occurred in the first and second trimester of gestation [44, 46, 47]. On the other hand, the lower multiplication of *N. caninum* in the maternal side of the placenta supports the hypothesis that caruncles act as a barrier, limiting not only parasite infection via reduced adhesion but also its multiplication. In experimental infections, comparisons between cytokine mRNA levels in separated maternal and foetal placental tissues showed that maternal tissue was the major source of most cytokines [48] and had a major lymphocyte cell infiltration, particularly in the maternal caruncle [7, 49], which may indicate that the maternal immune system was actively responding to the parasite. Moreover, early egress was observed in caruncular cells, which could be employed by the parasite as an escape mechanism to facilitate the dissemination of the parasite to the foetal part of the placenta, which has been demonstrated in this work to be the parasite’s preferential target cell, and, thus, allow vertical transmission of the parasite.

As mentioned above, the role of the parasite in the outcome of infection is also a determining factor. Processes involved in the lytic cycle, including parasite invasion and intracellular proliferation, are essential for the maintenance and multiplication of the parasite in vitro and for parasite survival and propagation in host tissues during the course of animal infection [1, 6]. In previous studies, several *N. caninum* isolates showed differences during the in vitro lytic cycle and more virulence than others in animal models, associated with higher abortion and transmission rates [16, 18, 20, 29, 31, 50]. In trophoblast cells, both isolates, described as “highly prolific” (Nc-Spain7) and “less prolific” (Nc-Spain1H) in previous studies [16], showed the same in vitro characteristics. In particular, the virulent isolate Nc-Spain7 demonstrated greater invasion, infection and proliferation rates than Nc-Spain1H in trophoblast cells. These differences may be explained by their biological diversity, as has been demonstrated in previous in vivo studies [16, 18, 20, 29, 32, 51, 52]. Nc-Spain7 showed a high neonatal mortality (95%) and vertical transmission rate (nearly 80%) in a pregnant BALB/c mouse model [31], as well as a percentage of abortion and vertical transmission as high as 100% in a bovine model [20, 53]. However, Nc-Spain1H showed a 100% offspring survival rate and a low vertical transmission rate (5%) in a pregnant mouse model [29], and no foetal death was observed in experimentally infected cattle [18]. The higher proliferation ability of Nc-Spain7 in trophoblast cells found in the present study may be responsible for the increase in the quantity of parasites reaching the foetal tissues and, consequently, for the enhancement of parasite burdens and pathology, ultimately resulting in foetal death and abortion. These results agree with those obtained in previous studies, where higher parasite burdens in the brain and placental tissues, a wider spread and greater severity of histopathological lesions and clinical signs were observed in animals experimentally infected with Nc-Spain7 [18, 20, 50]. However, in Nc-Spain1H-infected animals, less severe lesions were observed in placentas and maternal and foetal tissues [18], which may explain the absence of abortion. In terms of dissemination in vivo, isolates with low virulence could have a lower efficiency at crossing biological barriers.

More interestingly, contrary to our observation in trophoblast cells, the behaviour of both isolates was very similar in bovine caruncular cells. Differences between isolates were limited to a slightly higher infection rate by the virulent isolate Nc-Spain7, whereas adhesion, invasion and proliferation mechanisms were very similar for both isolates. This fact has also been observed in the phylogenetically-related protozoan *T. gondii*, where comparisons between three strains showed no significant differences in their capacity to infect human placental explants [11]. The comparable behaviour showed by different virulence isolates, together with the lower invasion, infection and proliferation rates found in caruncular cells, leads us to hypothesize that isolates may have been selected because of a low virulence in the maternal part of the placenta despite their differences in virulence traits in other host cells, including other placental cells such as trophoblasts. This reduced virulence in the caruncle may facilitate, on the one hand, evasion from maternal immunity and the placental damage caused by parasite multiplication, leading to the abortion. On the other hand, this behaviour may facilitate vertical transmission to the progeny, which is the main route of transmission for *N. caninum.* In fact, Nc-Spain7 and Nc-Spain1H isolates were obtained from healthy but congenitally infected calves, as described above.

## Conclusions

This is the first study where an in vitro model of *N. caninum* infection has been implemented in bovine placental cells. Our findings confirm a differential competency of two isolates of *N. caninum* with different virulence to proliferate in bovine trophoblast cells. However, bovine caruncular cells were the first cell line assayed where different virulence isolates showed similar invasion, adhesion and proliferation kinetics. The low replication of both isolates in the maternal side of the placenta may facilitate the evasion of the inmune response by the parasite, allowing their transplacental transmission. This fact may have constituted an evolutionary advantage for these isolates. Remarkably, limited parasite invasion and growth in caruncular cells suggest a putative barrier function for this cell type in the placenta, although early parasite egress may facilitate transmission to offspring.

Furthermore, our results confirm the role of foetal trophoblasts as target cells for *N. caninum*. Future research to determine the differences in surface receptors and cell junctions between both placental cell types are needed. In addition, studies focused on co-cultures of maternal and foetal cells may be helpful as a model to study parasite transport across the maternal epithelium as part of the bovine placental barrier. Finally, the existence of differences in local immunomodulation and cellular mechanisms that take place in the placenta infected by high- and low-virulence isolates of *N. caninum* should be investigated.

## Abbreviations

BCEC-1: Bovine caruncular epithelial cell line 1
BVD: Bovine viral diarrhea
CInfR: Cell infection rate
C_t_: Cycle threshold value
DMEM: Dulbecco’s Modified Eagle Medium
F3: Bovine placental trophoblast cell line
FCS: Foetal calf serum
hpi: Hours post-infection
MOI: Multiplicity of infection
PInvR: Parasite invasion rate
pInvR_T_: Total parasite invasion rate
qPCR: Real-time PCR
SD: Standard deviation
T_d_: Doubling time
TY_58h_: Tachyzoite yield at 58 h post-infection
